# Deeper Understanding of Appearance in Orofacial Clefts: A Structural Equation Model of the CLEFT-Q Appearance Scales

**DOI:** 10.1097/GOX.0000000000003806

**Published:** 2021-09-17

**Authors:** Conrad J. Harrison, Chris J. Sidey-Gibbons, Anne F. Klassen, Karen W. Y. Wong Riff, Dominic Furniss, Marc C. Swan, Jeremy N. Rodrigues

**Affiliations:** From the * Nuffield Department of Orthopaedics, Rheumatology and Musculoskeletal Sciences, University of Oxford, Oxford, United Kingdom; † MD Anderson Center for INSPiRED Cancer Care, the University of Texas, Houston, Tex.; ‡ Department of Pediatrics, McMaster University, Hamilton, Ontario, Canada; § Department of Plastic and Reconstructive Surgery, Hospital for Sick Children, Toronto, Ontario, Canada; ¶ The Spires Cleft Centre, John Radcliffe Hospital, Oxford University Hospitals, Oxford, United Kingdom; ∥ Department of Plastic Surgery, Stoke Mandeville Hospital, Buckinghamshire Healthcare NHS Trust, Aylesbury, United Kingdom.

## Abstract

**Background:**

The CLEFT-Q is a patient-reported outcome measure with seven scales measuring elements of facial appearance in cleft lip and/or palate. We built on the validated CLEFT-Q structural model to describe conceptual relationships between these scales, and tested our hypothesis through structural equation modeling (SEM). In our hypothesized model, the appearance of the nose, nostrils, teeth, jaw, lips, and cleft lip scar all contribute to overall facial appearance.

**Methods:**

We included 640 participants from the international CLEFT-Q field test. Model fit was assessed using weighted least squares mean and variance adjusted regression. The model was then refined through modification indices. The fit of the hypothesized model was confirmed in an independent sample of 452 participants.

**Results:**

The refined model demonstrated excellent fit to the data (comparative fit index 0.999, Tucker-Lewis index 0.999, root mean square error of approximation 0.036 and standardized root mean square residual 0.036). The confirmatory analysis also demonstrated excellent model fit.

**Conclusion:**

Our structural model, based on a clinical understanding of appearance in orofacial clefting, aligns with CLEFT-Q field test data. This supports the instrument’s use and the exploration of a wider range of applications, such as multidimensional computerized adaptive testing.

## INTRODUCTION

The CLEFT-Q is a patient-reported outcome measure (PROM) designed to measure the elements of health that matter most to people born with a cleft lip and/or palate. There is a growing body of evidence to support the CLEFT-Q’s validity,^1–4^ and it has been recommended as a major component of an international consensus-based core outcome set.^5^ The PROM contains 12 scales which assess different aspects of facial appearance, facial function, and cleft-related quality of life, plus an additional checklist that assesses eating and drinking. Each item contains three or four response options, for example, in the social function scale, the item “my friends accept me” has four response options: “never,” “sometimes,” “often,” and “always.”

**Fig. 1.**
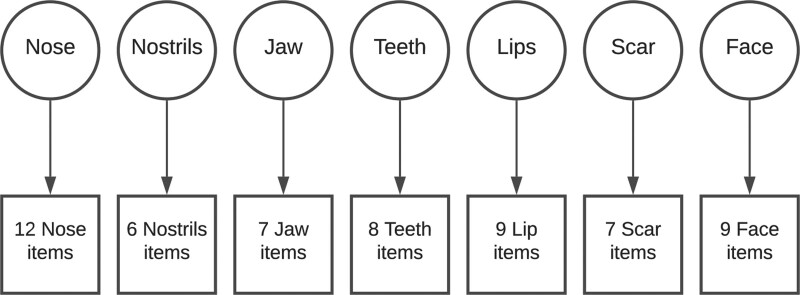
A schematic representing the existing structural model of CLEFT-Q appearance scales. Items have been grouped together for clarity.

The CLEFT-Q has been demonstrated to have seven independently-functioning appearance scales that each measure one aspect of facial appearance: nose, nostrils, jaw, teeth, lips, (cleft lip) scar, and face (which measures overall facial appearance).^2^ Under this validated conceptual framework (depicted in Fig. 1), items in a scale reflect a single construct. In this model, we might know that a respondent scores poorly on the appearance of their nose, lips, teeth, scar, and jaw, but we would not be able to use these scores to predict their response to items in the Face scale, such as “how much do you like how your face looks in photographs?”

Based on the clinical opinion, it is plausible that the appearance of a person’s nose, nostrils, jaw, teeth, lips, and cleft lip scar might contribute to the overall appearance of their face, along with other unmeasured constructs (eg, the appearance of the eyes and ears). It is also likely that clinically related scales have probabilistically related scores (eg, nose and nostril differences in orofacial clefts are related by etiology and treatment).

These conceptual models represent different approximations to a ground truth that cannot be determined fully. Thus, if each meet appropriate assumptions and display acceptable model fit, they might each be used for distinct applications. For example, probabilistic relationships between CLEFT-Q scales may support the development of shorter, more personalized, cleft assessments through multidimensional computerized adaptive testing (CAT).

If statistical relationships in CLEFT-Q scale scores reflect our clinical understanding of cleft appearance this would further support the instrument’s use. It is possible to investigate these relationships through structural equation modeling (SEM), a branch of psychometrics that models the relationship between indicators (items in a questionnaire) and factors (health constructs) through regression equations.^6^ SEM is described extensively within the psychological literature, but its application to surgical PROMs is relatively novel. In this study, we use SEM to test and refine a conceptually plausible model of the seven CLEFT-Q appearance scales.

## METHODS

### Conceptual Model

**Fig. 2.**
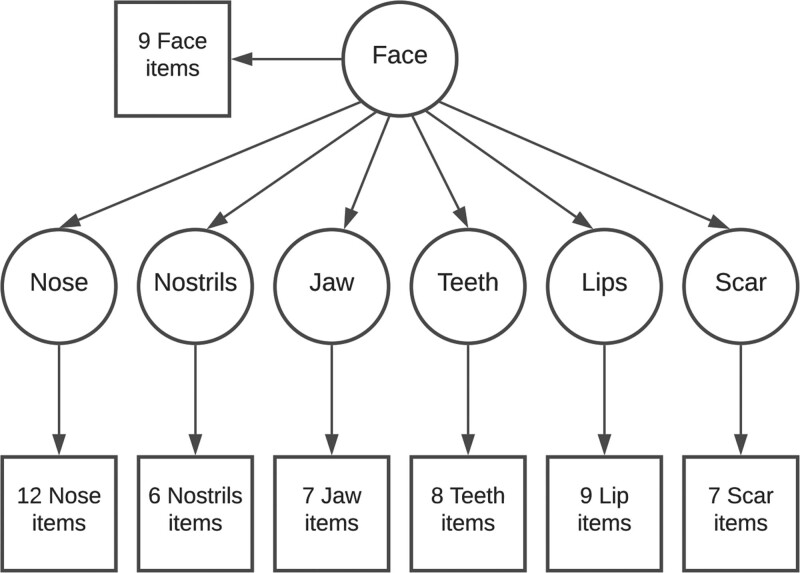
Hypothesized structural equation model. Items have been grouped together for clarity.

Figure 2 demonstrates our hypothesized model. In this model, there are six first-order factors (appearance of nose, nostrils, jaw, teeth, lips, and scar). Each of these factors is measured by the items that make up those respective scales. There is one second-order factor, appearance of the face, which can be measured by items from the Face scale, and to some extent by the first-order factors.

### Study Participants

To test our hypothesis, we used item responses from the CLEFT-Q field test. This was a prospective international study which recruited from October 2014 to November 2016 and involved 2434 participants from 30 centers in 12 countries. Participants were 8 to 29 years of age and had been diagnosed with either a cleft lip, cleft palate, cleft lip, and alveolus or cleft lip and palate. A detailed description of the CLEFT-Q study participants has been published elsewhere.^2^

### Software

We conducted our analysis using R v 4.0.0 with the following packages: foreign v 0.8-78, psych v 1.9.12.31 mice v 3.8.0, dplyr v 0.8.5, lavaan 0.6-5.^7^

### Exploratory Factor Analysis

For the purposes of this study, we did not assume structural validity of the CLEFT-Q. Before undertaking any confirmatory analyses, we performed an exploratory factor analysis (EFA) to identify factors measured by the 58 CLEFT-Q appearance items. This was conducted using the *psych* package in *R*. Of the 2434 CLEFT-Q field test participants, we included 1092 who had fewer than three missing responses (ie, all participants who had fewer than 5% of their response data missing). We then used a single iteration of multivariate imputation with chained (MICE) and a proportional odds model to replace 241 missing responses, as described for factorial analyses.^8,9^ Following this, we identified 108 outliers based on Mahalanobis distance. When the analysis was repeated without these outliers there were no meaningful differences in model fit (without outliers: comparative fit index (CFI) 0.945, Tucker-Lewis index (TLI) 0.928, root mean square error of approximation (RMSEA) 0.049, standardized root mean square residual (SRMR) 0.020; cf, with outliers: CFI 0.946, TLI 0.929, RMSEA 0.048, SRMR 0.020). Hence, we have included them in the reported analysis.

Collinearity was assessed with a correlation plot. Other assumptions were tested in a linear regression against a randomly generated dataset with a chi-squared distribution and four degrees of freedom. Correlation adequacy was assessed with Bartlett’s test, and sampling adequacy was assessed with the Kaiser–Meyer–Olkin test.

A scree plot, parallel analysis, and Kaiser criterion analysis (with a threshold of 0.70) were used to determine the number of factors to use. Factor analysis was performed using weighted least squares and direct oblimin rotation, assuming seven factors, aligning with the original conceptual model.

### Sample Size

Following EFA, we randomly resampled participants (without replacement) from the whole dataset for SEM analysis. There is no consensus on the correct sample size for SEM analyses.^10^ Complex models, incomplete datasets, nonnormality, ordinal data, a large indicator-to-factor ratio, and covariance between factors or between indicators are all features to support a large sample size. However, SEM fit test statistics are sensitive to overpowering and a large sample size can increase the chance of a type I error. This is particularly true for the chi-square model fit statistic.^11^

Typically, SEM analyses involve 200–500 participants. One heuristic method is to include 10 participants per indicator. Another recommended technique is to include 10 participants per parameter estimate.^10^ Our model contains 58 indicators (items) and 64 parameter estimates, represented by the arrows in Figure 2 (one per item, plus six between the first-order factors and the higher-order factor). Given the large number of indicators in our model and the nature of our ordinal response data, we chose a sample size of 640 and accepted the risk of a type I error.

As with our EFA, we included outliers defined by Mahalanobis distance in the SEM sample. When the analysis was repeated without outliers, there were no meaningful differences to model fit statistics or parameters.

### Missing Data

We performed a missing data analysis of the CLEFT-Q field test results and excluded 1342 participants that were missing more than two responses to the 58 items included in our analysis (ie, we included participants who were missing <5% of responses, as with the EFA, in keeping with recommendations for factorial analyses).^9^ Of these, 686 were missing exactly seven items and 171 were missing exactly 14 items. This is because in the CLEFT-Q field test, the Jaw scale (seven items) was only administered to respondents aged 12 years and older, and the Scar scale (also seven items) was only administered to respondents who were born with a cleft lip, representing populations that would be likely to complete the scales in a real-world setting.^2^ In total, 958 participants had not completed any Jaw scale items, and 715 participants had not completed any Scar scale items.

Following exclusion, we randomly selected 640 of 1092 participants with 146 missing responses and 35,814 complete responses. There was no obvious trend in the items that were missed. These missing responses were imputed using a single iteration of MICE with a proportional odds model, which is recommended for handling missing item responses.^9^

### Assumption Testing

The assumptions of normality and homoscedasticity were retested in this sample using the same techniques described for the EFA. Fitted versus residual scatter plots suggested linearity and heteroscedasticity, and a Q-Q plot suggested nonnormality (**See Appendix, Supplemental Digital Content 1**, which displays (A) Q-Q plot for the EFA sample, (B) fitted versus residuals plot for the EFA sample, (C) parallel analysis scree plot, (D) Q-Q plot for the primary SEM sample, and (E) fitted versus residuals plot for the primary structural equation modeling sample; **http://links.lww.com/PRSGO/B777**). We therefore used weighted least squares mean and variance adjusted (WLSMV) regression in the SEM, which is robust for ordinal data with large sample sizes, even when assumptions of normality and homoscedasticity are violated.^12,13^

### Assessment of Model Fit

We applied five tests of model fit that compared our model to a null model (which assumed factor independence): model Chi-square (χ^2^), CFI, TLI, RMSEA, and SRMR. We considered values of χ^2^ *P* greater than 0.05 or greater, TLI 0.950 or greater, CFI 0.950, RMSEA less than 0.060, and SRMR 0.080 or less to indicate good model fit.^6^

### Model Refinement

Once model fit was tested, we considered suggested alterations accompanied by a modification index (MI) which estimates the drop in the χ^2^ test statistic that would follow the modification.^14^ We examined alterations associated with relatively large modification indices (>80), and iteratively included these in a refined model if they made sense conceptually. Modifications were made in order of MI size, with the modification associated with the largest improvement in model fit made first. We tested each iteration of the refined model using the five fit statistics described above.

### Confirmatory Analysis

In a repeat analysis, we re-evaluated the model with the 452 participants who had fewer than three missing responses and were not previously sampled.

## RESULTS

### Exploratory Factor Analysis

Table 1 shows the clinical and demographic details of the 1092 included participants. The correlation plot revealed no perfect (r > 0.99) collinearity between items. Inspection of fitted versus residual scatter plots suggested linearity and heteroscedasticity, and a Q-Q plot suggested nonnormality. Bartlett’s test confirmed correlation adequacy (χ^2^(1653) = 61883, *P* < 0.01) and the Kaiser–Meyer–Olkin test confirmed sampling adequacy (overall measure of sampling adequacy = 0.98). A scree plot and Kaiser criterion analysis suggested seven factors, whereas parallel analysis suggested eight.

**Table 1.** Demographics and Clinical Details of 1092 Included Participants

Age (years)	Median (IQR)	16 (5)
Gender	Male	634
	Female	457
	Missing	1
Country	Australia	6
	Canada	224
	England	133
	Ireland	73
	USA	142
	Netherlands	100
	India	96
	Sweden	41
	Turkey	44
	Columbia	117
	Chile	67
	Spain	49
Cleft type	Cleft lip	139
	Cleft lip and alveolus	116
	Cleft lip, alveolus and palate	837
Lip type	Unilateral	782
	Bilateral	304
	Missing	6

IQR, interquartile range.

Two items demonstrated split-loadings (pattern coefficient > 0.30): Face item 8 loaded onto the Nose factor (0.42) more than the Face factor (0.31) and Nose item 12 loaded onto the Nostril factor (0.39) as well as the Nose factor (0.43). The Nose and Nostril factors correlated by 0.74 and the Lips and Scar factors correlated by 0.62. The model had moderate fit (CFI 0.946, TLI 0.929, RMSEA 0.048, and SRMR 0.02).

### Hypothesized Model Fit

Model fit statistics for our hypothesized model are presented in Table 2. The model demonstrated excellent model fit in all indices except χ^2^.

**Table 2.** Refined Model Fit Statistics

	χ^2^ (*P*)	CFI	TLI	RMSEA	SRMR
Threshold	>0.05	≥0.95	≥0.95	<0.06	≤0.08
Hypothesized model	0.000	0.998	0.998	0.043	0.039
Refined model	0.000	0.999	0.999	0.036	0.036

### Model Refinement

Table 3 describes the refinements we made to our model, based on MI. Each modification was assessed for clinical relevance before being iteratively incorporated. Model fit improved with each iteration.

**Table 3.** Model Refinements

Modification	Rationale
Teeth factor is measured by Face item 6	Face item 6 relates to the appearance of the face when the respondent is smiling. Dissatisfaction with the appearance of one’s teeth may contribute to a poor score on this item
Nose factor is measured by Face item 5	Face item 5 relates to facial symmetry. The relationship between facial symmetry and nasal appearance is well established, and facial symmetry has previously been used as a measure of nasal appearance in orofacial cleft research^15^
Face item 1 correlates with Face item 2	Face item 1 asks about facial appearance at its “best” and Face item 2 asks about facial appearance when the respondent is ready to go out “like to a party”. These items are conceptually very similar, and it is plausible that responses share a high covariance
Face item 8 correlates with Nose item 11	Face item 8 relates to the appearance of the face from a side profile, Nose item 11 relates to the appearance of the nose from a side profile

### Refined Model Fit

**Fig. 3.**
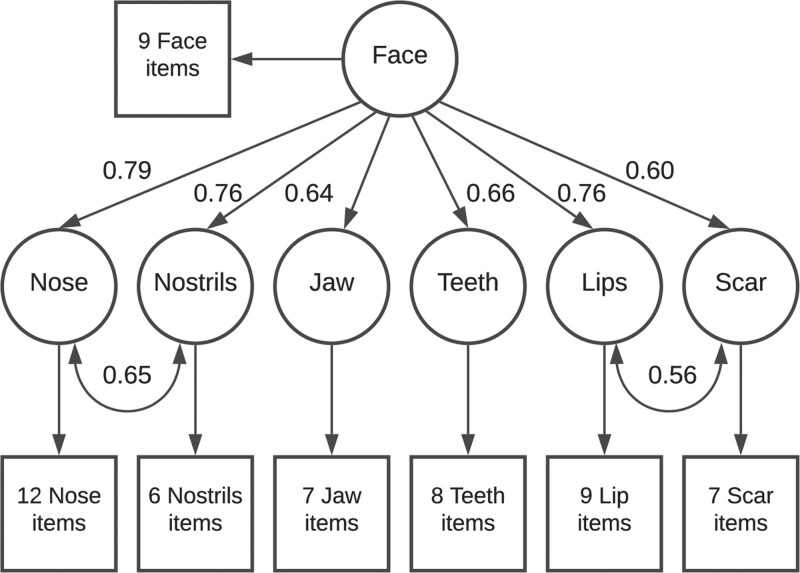
Simplified structural equation schematic for the refined model. Straight arrows are labeled with standardized regression (WLSMV) coefficients. Curved arrows represent residual covariance between factors.

Refined model fit statistics are displayed in Table 2. Each of the first-order factors loaded information onto the Face factor, with standardized regression coefficients ranging from 0.60 to 0.79. Refined model parameters are available in Supplemental Digital Content 2. (**See Appendix, Supplemental Digital Content 2**, which contains (A) structural equation model parameters calculated with outliers included, and (B) structural equation model parameters calculated with outliers excluded; **http://links.lww.com/PRSGO/B778**.) Figure 3 demonstrates a selection of key model parameters.

### Repeat Analysis

The hypothesized model also achieved excellent fit in the confirmatory analysis (CFI 0.998, TLI 0.998, RMSEA 0.038, and RMSR 0.043). Only one modification was adopted during the confirmatory analysis. This was “Face item 1 correlates with Face item 2.” After making this modification the model fit statistics were CFI 0.998, TLI 0.998, RMSEA 0.036, and RMSR 0.043.

## DISCUSSION

### Principal Findings

In our EFA, we identified seven factors measured by the CLEFT-Q appearance items. This result corroborates previous research into the structural validity of the CLEFT-Q.^2^ The EFA suggested a correlation between scores in the Nose and Nostrils scales, and between scores in the Lip and Scar scales. This is supported by our practical understanding of appearance in orofacial clefting, as these constructs are related clinically.

Our hypothesized model demonstrated excellent fit statistics, except for the χ^2^ *P* values. The χ^2^ statistic is sensitive to small model inaccuracies in large sample sizes, as used in this study, and is almost always statistically significant in models with 400 or more cases.^11^ Thus, they may represent the type I errors which were anticipated a priori.

We adopted four modifications to the hypothesized model, based on modification indices and clinical judgment. This could be early evidence that some items in the CLEFT-Q can measure more than one latent trait. For example, our model had improved fit when an item about smiling (Face item 6) measured both the appearance of the face and the appearance of the teeth.

### Strengths and Limitations

We selected a large sample size (640) to accommodate these data in a complex model design, and this is likely to have led to a type I error in the χ^2^ fit test statistic. We selected a regression method that is robust to violations of normality and homoscedasticity, and by incorporating a repeat analysis, we were able to demonstrate excellent fit statistics (CFI, TLI, RMSEA, and RMSR) for our hypothesized model in two independent samples.

We included model refinements that were data-driven but also supported by clinical reasoning. Although modification indices may play an important role in avoiding the misspecification of complex second-order SEMs as bifactor models,^16^ iteratively improving model fit does not necessarily improve the usefulness of a model. In 2001, Ullman likened post-hoc SEM modifications to eating salted peanuts because “one is never enough.”^6^ Our model modifications should be interpreted cautiously, as the only one we reproduced in our repeat analysis was the correlation of Face item 1 and Face item 2.

### Study Implications

This article supports the construct validity of the CLEFT-Q appearance scales. Our findings imply that the CLEFT-Q appearance scales measure what clinicians might expect them to measure, in the way we expect them to be measured, that is, clinically correlated factors have correlated scores, and appearance of individual facial features contribute to the overall appearance of the face.

These findings could improve the efficiency of CLEFT-Q item administration. For example, if we know that Nose scores and Nostrils scores correlate, and that a respondent has attained a high score in the Nose scale, it may be more appropriate to start a Nostril scale CAT with a question targeted towards respondents with a higher Nostrils score. Future work will determine whether the accuracy and efficiency of the CLEFT-Q CAT scales^17^ could be improved with multidimensional item response theory.

## CONCLUSIONS

We have proposed a conceptually-driven, second-order structural model for the CLEFT-Q appearance scales, and demonstrated excellent model fit among participants of the CLEFT-Q field test, in two independent samples. This article provides additional evidence to support the validity of the CLEFT-Q and enables future work into the application of multidimensional CAT to the PROM.

## Supplementary Material


