# Supplementary material for: Deeper Understanding of Appearance in Orofacial Clefts: A Structural Equation Model of the CLEFT-Q Appearance Scales
Source: Plast Reconstr Surg Glob Open. 2021 Sep 17;9(9):e3806. doi: 10.1097/GOX.0000000000003806 (PMC8447998; doi:10.1097/GOX.0000000000003806)
Supplement: Supplementary file 1 [file gox-9-e3806-s001.pdf]

Deeper understanding of appearance in orofacial clefts: a structural equation model of the CLEFT-Q appearance scales

Supplemental Digital Content 1. Supplementary figures demonstrating results of regression assumption testing and parallel analysis.

1A. Q-Q plot for the exploratory factor analysis sample.

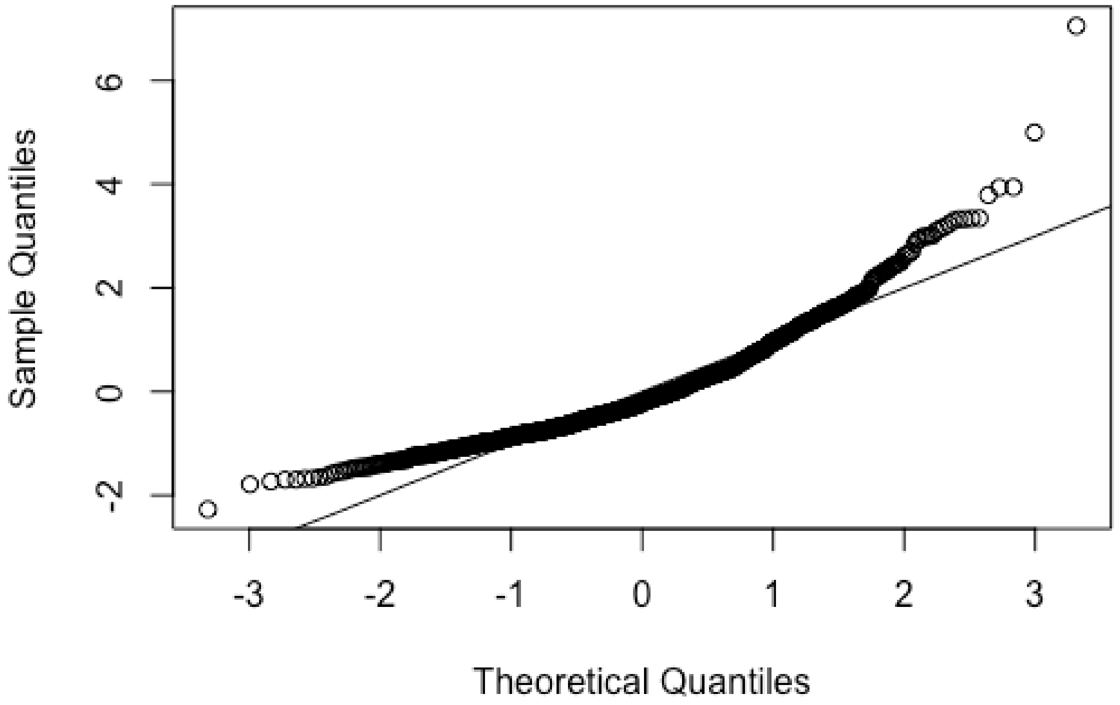

Deeper understanding of appearance in orofacial clefts: a structural equation model of the CLEFT-Q appearance scales

1B. Fitted vs residuals plot for the exploratory factor analysis sample

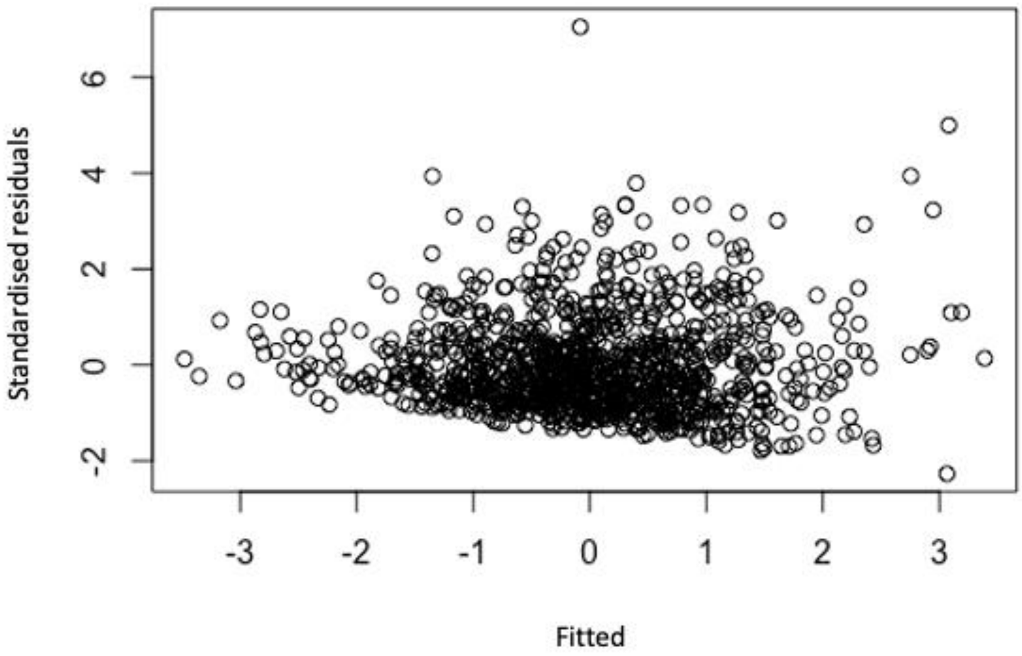

.

1C. Parallel analysis scree plot.

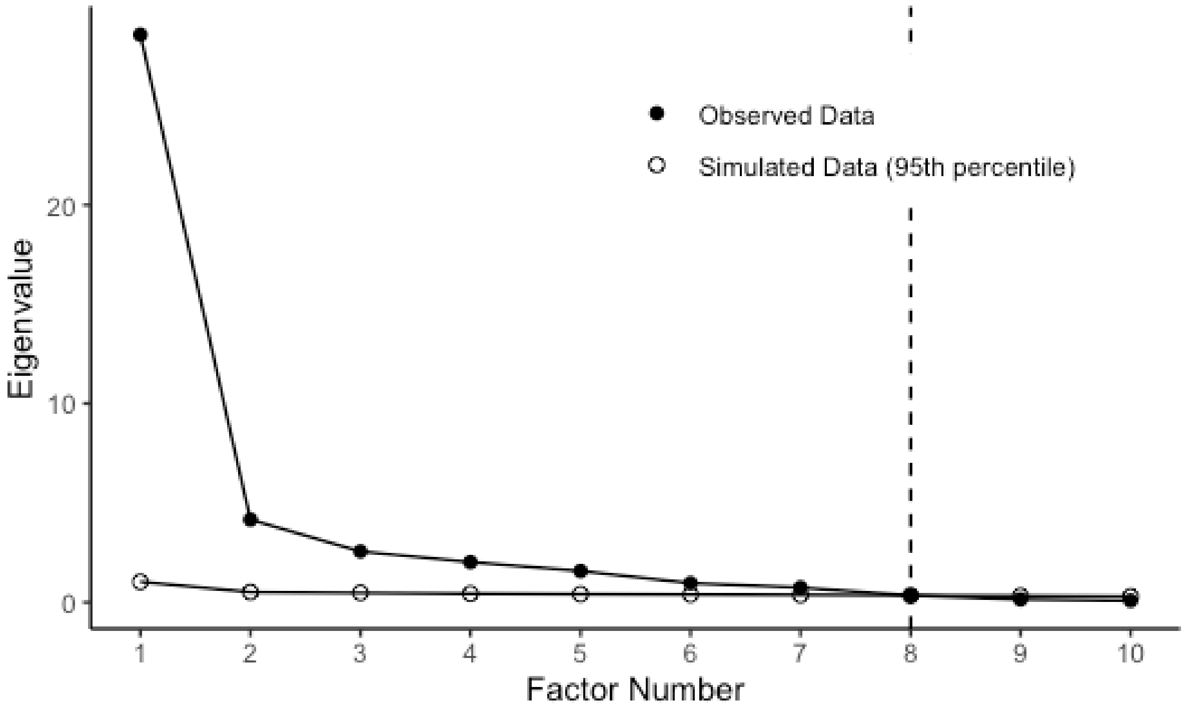

1D. Q-Q plot for the primary structural equation modelling sample.

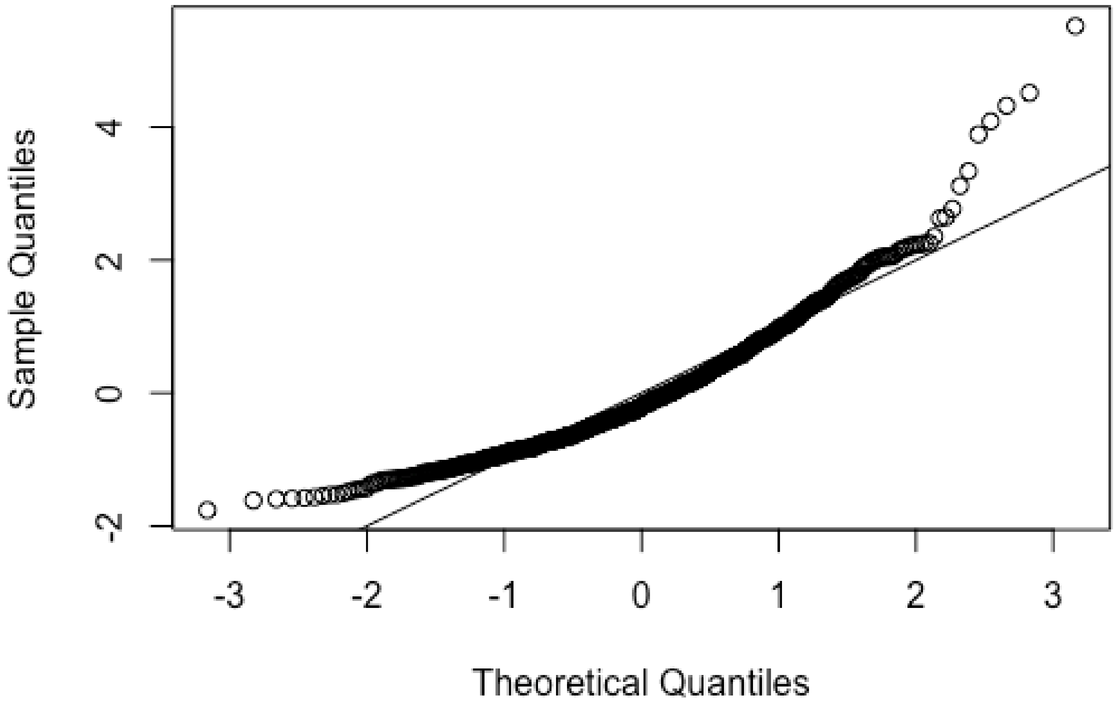

1E. Fitted vs residuals plot for the primary structural equation modelling sample.

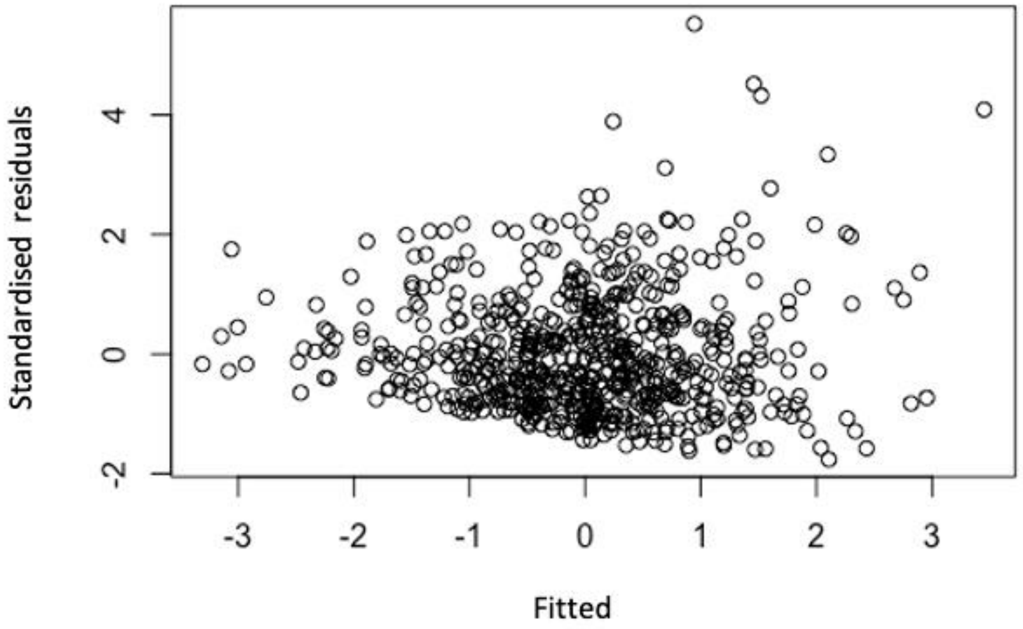

Deeper understanding of appearance in orofacial clefts: a structural equation model of the CLEFT-Q appearance scales
