# Supplementary material for: Deeper Understanding of Appearance in Orofacial Clefts: A Structural Equation Model of the CLEFT-Q Appearance Scales
Source: Plast Reconstr Surg Glob Open. 2021 Sep 17;9(9):e3806. doi: 10.1097/GOX.0000000000003806 (PMC8447998; doi:10.1097/GOX.0000000000003806)
Supplement: Supplementary file 2 [file gox-9-e3806-s002.pdf]

SDC 2. Structural equation model parameters

First order factor loadings

Factor	Indicator	Regression coefficient	Standardized regression coefficient	Standard Error	Wald statistic
	Face 1	1	0.849		
	Face 2	0.928	0.788	0.02	46.536
	Face 3	0.812	0.689	0.027	29.784
	Face 4	0.964	0.818	0.023	42.062
Face	Face 5	0.502	0.426	0.061	8.24
	Face 6	0.814	0.691	0.03	26.962
	Face 7	1.025	0.87	0.022	45.838
	Face 8	0.956	0.811	0.023	42.124
	Face 9	1.035	0.879	0.022	47.455
	Nose 1	1	0.869		
	Nose 2	1.042	0.906	0.014	73.225
	Nose 3	1.036	0.901	0.014	76.222
	Nose 4	0.95	0.826	0.015	65.25
	Nose 5	1.049	0.912	0.015	72.09
	Nose 6	0.952	0.828	0.018	53.102
Nose	Nose 7	1.006	0.875	0.015	69.311
	Nose 8	1.046	0.909	0.015	69.475
	Nose 9	1.03	0.895	0.014	72.823
	Nose 10	1.003	0.872	0.015	69.024
	Nose 11	1.015	0.883	0.014	72.874
	Nose 12	0.973	0.846	0.018	54.767
	Face 5	0.407	0.354	0.057	7.174
	Nostrils 1	1	0.964		
	Nostrils 2	1.003	0.967	0.006	163.055
	Nostrils 3	0.958	0.924	0.008	122.068
Nostrils	Nostrils 4	0.954	0.92	0.008	113.145
	Nostrils 5	0.963	0.928	0.008	119.511
	Nostrils 6	0.953	0.919	0.008	114.147
Jaw	Jaw 1	1	0.955		

	Jaw 2	1.001	0.956	0.007	152.286
	Jaw 3	1.005	0.96	0.006	169.206
	Jaw 4	0.982	0.938	0.008	126.343
	Jaw 5	0.973	0.929	0.008	129.045
	Jaw 6	1.006	0.961	0.006	167.333
	Jaw 7	0.971	0.928	0.008	129.041
	Teeth 1	1	0.89		
	Teeth 2	0.954	0.849	0.02	46.639
	Teeth 3	1.039	0.924	0.019	56.129
	Teeth 4	1.049	0.933	0.019	55.551
Teeth	Teeth 5	0.912	0.811	0.022	41.203
	Teeth 6	1.006	0.895	0.02	50.029
	Teeth 7	1.046	0.93	0.018	56.658
	Teeth 8	0.921	0.819	0.024	38.305
	Face 6	0.235	0.209	0.031	7.653
	Lips 1	1	0.915		
	Lips 2	0.97	0.888	0.012	81.061
	Lips 3	1.012	0.926	0.011	92.02
	Lips 4	1.032	0.945	0.011	93.164
Lips	Lips 5	1.01	0.924	0.011	92.615
	Lips 6	0.944	0.864	0.013	72.069
	Lips 7	1	0.915	0.011	92.901
	Lips 8	0.939	0.859	0.014	68.022
	Lips 9	0.997	0.913	0.012	82.538
	Scar 1	1	0.895		
	Scar 2	1.065	0.954	0.011	101.221
	Scar 3	1.055	0.945	0.01	102.988
Scar	Scar 4	1.072	0.96	0.011	101.22
	Scar 5	1.034	0.926	0.011	91.325
	Scar 6	1.061	0.95	0.011	95.845
	Scar 7	1.059	0.948	0.011	100.143

Higher (Face) factor loadings

First order factor	Regression coefficient	Standardized regression coefficient	Standard Error	Wald statistic
Nose	0.838	0.818	0.021	39.485
Nostrils	0.863	0.76	0.022	38.511

	Jaw	0.702	0.624	0.027	25.607
	Teeth	0.664	0.633	0.026	25.597
	Lips	0.829	0.769	0.022	36.921
	Scar	0.661	0.626	0.027	24.738

Indicator covariance

First indicator	Second indicator	Covariance	Standardized covariance	Standard Error	Wald statistic
Face 1	Face 2	0.198	0.608	0.02	10.041
Face 8	Nose 11	0.172	0.624	0.014	12.326

Factor covariance

First factor	Second factor	Covariance	Standardized covariance	Standard Error	Wald statistic
Nose	Nostrils	0.209	0.668	0.016	12.909
	Jaw	0.045	0.121	0.015	3.124
	Teeth	0.029	0.083	0.014	2.091
	Lips	0.058	0.2	0.011	5.293
	Scar	0.076	0.218	0.013	6.01
Nostrils	Jaw	0.041	0.088	0.018	2.324
	Teeth	0.03	0.07	0.017	1.809
	Lips	0.106	0.288	0.014	7.603
	Scar	0.113	0.258	0.015	7.571
Jaw	Teeth	0.193	0.376	0.019	10.377
	Lips	0.14	0.321	0.018	7.789
	Scar	0.194	0.372	0.02	9.683
Teeth	Lips	0.11	0.273	0.015	7.457
	Scar	0.124	0.258	0.017	7.142
Lips	Scar	0.203	0.498	0.018	11.58

Indicator variance

Indicator	Variance
Face 1	0.28
Face 2	0.379
Face 3	0.525
Face 4	0.33
Face 5	0.446

Face 6	0.297
Face 7	0.243
Face 8	0.342
Face 9	0.228
Nose 1	0.244
Nose 2	0.179
Nose 3	0.188
Nose 4	0.317
Nose 5	0.168
Nose 6	0.315
Nose 7	0.235
Nose 8	0.173
Nose 9	0.198
Nose 10	0.239
Nose 11	0.221
Nose 12	0.284
Nostrils 1	0.071
Nostrils 2	0.066
Nostrils 3	0.147
Nostrils 4	0.154
Nostrils 5	0.139
Nostrils 6	0.156
Jaw 1	0.087
Jaw 2	0.086
Jaw 3	0.078
Jaw 4	0.12
Jaw 5	0.136
Jaw 6	0.077
Jaw 7	0.139
Teeth 1	0.209
Teeth 2	0.28
Teeth 3	0.146
Teeth 4	0.13
Teeth 5	0.342
Teeth 6	0.199
Teeth 7	0.135
Teeth 8	0.329

Lips 1	0.163
Lips 2	0.212
Lips 3	0.142
Lips 4	0.108
Lips 5	0.146
Lips 6	0.253
Lips 7	0.163
Lips 8	0.262
Lips 9	0.167
Scar 1	0.198
Scar 2	0.09
Scar 3	0.107
Scar 4	0.079
Scar 5	0.142
Scar 6	0.097
Scar 7	0.102

Factor variance

Factor	Variance	Standardized variance	Standard Error	Wald statistic
Face	0.72	1	0.025	28.992
Nose	0.25	0.331	0.017	14.851
Nostrils	0.393	0.423	0.023	17.051
Jaw	0.558	0.611	0.025	21.956
Teeth	0.474	0.599	0.025	18.603
Lips	0.342	0.409	0.021	16.662
Scar	0.487	0.608	0.023	21.274

These parameters were calculated with outliers excluded
